# Adolescents’ experiences with the food selection at the sports arena in the area of Oslo, Norway: a focus group study

**DOI:** 10.1017/S1368980024000181

**Published:** 2024-01-19

**Authors:** Lisa Garnweidner-Holme, Pauline Alise Leganger Wattenberg, Therese Fostervold Mathisen, Mari Charlotte Wik Myhrstad

**Affiliations:** 1 Department of Nursing and Health Promotion, Faculty of Health Sciences, P.O. 4, St. Olavs Plass, Oslo Metropolitan University, Oslo 0130, Norway; 2 Faculty of Health, Welfare, and Organization, Østfold University College, Halden 1575, Norway.

**Keywords:** Sports arenas, Handball, Football, Healthy food, Adolescence, Qualitative research

## Abstract

**Objective:**

To investigate adolescents’ experiences with the food selection at the sport arena.

**Design:**

Four focus group interviews were conducted with 4–6 participants each. Interviews were recorded and transcribed verbatim. The transcripts were coded in NVivo and the analysis was guided by thematic analysis.

**Setting:**

Adolescents from handball and football clubs in Oslo and Viken, Norway, participated in the study.

**Participants:**

A total of nine boys (11–14 years old) and ten girls (11–14 years old) participated in the study.

**Results:**

We identified four main themes: interest for healthy food; experiences with the food selection at the sports arena; factors influencing participants’ food choices at the sports arena and expectations related to a healthy food selection at the sports arena. Adolescents across the focus groups experienced the food selection at the sports arena as unhealthy. Price, marketing and availability of unhealthy food were important factors that influenced their food choices at the sports arena. The trainer appeared to motivate the participants to eat healthy.

**Conclusions:**

Participants wished for a healthier food selection at the sports arena. Cost of food emerged as a factor that influenced their food choices. Our study also indicates that marketing of unhealthy food and beverages should be restricted, to influence adolescence food choice towards healthier alternatives.

Adolescence (10–19 years) is an important period for the establishment of healthy behaviours and for laying the foundations of good health^(1)^. Sport is integral to many adolescents’ lives, and those who frequently play sport spend considerable amount of time in these activities outside of school. Thus, sports arenas may be important to promote health and stimulate positive healthy behaviours^(2–6)^. However, sports arenas are often characterised by the unhealthy food on offer, which includes a wide range of energy-dense, nutrient-poor, processed items that are quick to prepare and inexpensive to provide^(2,7–11)^. Studies from Switzerland and the USA have revealed how convenience foods that are high in calories and sugar are readily available in youth sports settings^(12,13)^. A cross-sectional study on 301 sports clubs in Norway revealed that a majority offered sugar-rich drinks, sweetened bakery products and processed meat products (in review).

Past studies have identified barriers to healthy food selection at sports arenas^(4,8,14,15)^, such as the limited availability of healthy options and the presence of unhealthy food and beverage sponsorships^(16)^. The provision of food at sports arenas varies as per the type of arena and the country in which it is located^(3,8,17)^. In Norway, football and handball arenas have kiosks that are only occasionally open, which depends on the size of the club. The kiosk’s profits are one of the most important sources of income for sports clubs^(18)^.

A systematic review that investigated children’s and parents’ opinions on sport-related food environments suggested that they did not consider the environment to be conducive or supportive of children’s healthy food behaviours^(3)^. While increased profit and reduced food waste are arguments supporting the availability of highly processed and less healthy foods at the sports arena, parents were also often found to value unhealthy food regardless of how contradictory they were to healthy food behaviours. They believed that it supported the social environment of the arena and served as a reward for the children^(19)^. Despite sports arenas being considered important avenues for the development of healthy behaviours in adolescents^(2,3,20)^, little is known about adolescents’ experiences with the food selection available at such arenas.

The aim of this study was to investigate adolescents’ experiences with the selection of food available at sports arenas. Football and handball clubs were chosen because both are popular in Norway and their food environments remain consistent year-round, irrespective of season or weather.

## Methods

### Study setting

The study was conducted among adolescence in the area of Oslo, Norway, who were active in football or handball, the most popular activities in Norway. At Norwegian sport arenas (i.e. an indoor sport hall including outdoor sporting facilities, often a property of the regional sport federation or the municipality), sports clubs often hold kiosks. The kiosk sales are often arranged on a volunteering basis, where parents help out with food preparation and sale during opening hours. The chosen clubs represent a variety of location-specific socio-economic population samples and club membership sizes. The eastern region of Oslo is characterised by a higher immigrant and lower socio-economic population than the western region of the city.

### Participants

Nine boys (11–14 years old) and ten girls (11–14 years old) participated in the study. Table 1 provides background information on the focus group’s participants and the sports clubs they belong to. Participants’ parents provided written informed consent. The study was approved by (blinded information). Recruitment was carried out until we observed informational power^(21)^. The study was conducted as per the COREQ checklist^(22)^.

**Table 1:** Background information of the focus group’s participants and the sports clubs

Focus group nr.	Number of participants	Gender	Age (years)	Type of sport	Region of the sports club
1	4	Male	11–12	Football	Viken (West of Oslo)
2	5	Male	13–14	Football	Oslo (East)
3	4	Female	11–12	Handball	Oslo (South)
4	6	Female	13–14	Handball	Oslo (East)

### Recruitment

The second author recruited the participants from the personal network of the last author and by contacting the trainers at sport clubs who participated in our previous study^(19)^. The trainers provided contact information of participants’ parents to the second author, who scheduled the interviews. The second author contacted also trainers of other sport clubs (approximately 10); however, they did not have the time to help with the recruitment for this study. The recruitment process was conducted until we had achieved sufficient informational power, depending upon the quality of the interviews and the aim of the study^(21)^.

### Data collection

The project team developed a semi-structured interview guide based on the interview guide from a previous study among parents and club managers^(19)^. The project team consisted of experts in (sports) nutrition, public health nutrition and qualitative methods. The first author is professor in nutrition communication and experienced in qualitative methods. The second author (BSc), master student in Public Health Nutrition, conducted the four focus group interviews between October 2022 and January 2023. The second author, supervised by the first author, pilot tested the interview guide. The pilot-test only led to minor changes in the guide, for example, participants were asked what they considered as healthy food. Thus, we included the pilot interview in the final analysis for this study. The interviews began with a short introduction to the aim of the study and the background of the interviewer. After the introduction, open-ended questions were presented to facilitate discussions. The main themes in the interview guide were: (1) general perceptions towards healthy food at the sports’ arena; (2) experiences with the food selection at the sports’ arena and (3) suggestions for efforts to secure a healthy food selection at the sports arena. Compared with the interview guide targeting a younger age group^(19)^, the interview guide for this study also addressed that adolescence could buy food on their own. The participants were informed that they could withdraw their consent at any time without any reason. The interviewer did not knew the participants prior to the interview. One interview was conducted at a participant’s home, and the remaining three interviews were conducted at the clubhouse of the arena. The interviews lasted between 19 and 40 min. All interviews were digitally audio recorded and transcribed verbatim by the second author. The interviewer made field notes during the interview. The second and first author, read through the transcriptions to secure their trustworthiness. Participants were informed that they could read the transcripts of the interviews in the information sheet of this study. However, none of the participants asked to read the transcripts. All the interviews were conducted in Norwegian. Subthemes, overarching themes and quotes were translated into English by the authors for the purpose of this study.

### Data analysis

The analysis was guided by Braun and Clarke’s thematic analysis and included the following steps^(23)^: (1) the first and second author repeatedly read the transcripts to become familiar with the interviews; (2) the second author generated initial codes (words or short phrases from the transcripts) that were relevant to the research questions; the initial codes were discussed with the first author until consensus was achieved; (3) the first and the second author organising codes into subthemes; (4) the first and the second author arranged subthemes into overarching themes; the overarching themes were discussed with the last author until consensus was achieved and (5) the first and the second author defined and named the final themes. The qualitative software program NVivo (12.1) was used to identify the codes and systematise the subthemes.

## Results

Table 2 summarises the main themes and subthemes related to participants’ experiences with and expectations of the food selection available at the sports arena.

**Table 2:** Summary of main themes and subthemes related to participants’ experiences with and expectations of the food selection at the sports arena

Main themes	Interest for healthy food	Experiences with the food selection at the sports arena	Factors influencing participants’ food choices at the sports arena	Expectations related to a healthy food selection at the sports arena
**Subthemes**	Importance of healthy eating	Lack of healthy food and beverages	Availability of healthy food	Expects healthy food at the sports arena
	Definition of a healthy diet	Acceptance of the food selection	High price	Club/kiosk leaders and tournament organisers are responsible for a healthy food selection
	Motivation for healthy eating for physical performance	Adolescents are not the target group for sales at the kiosks	Parents and coaches influence adolescents’ food choices	
			Marketing of unhealthy food	
			Buy the same food as their peers	

### Interest for healthy food

Participants said that a healthy diet was important for them. They appeared to be interested in eating healthy. Participants defined a healthy diet as consisting of nutritious food that provides energy, is satiating and is low in fat. In this context, many described a varied diet that included fruits, vegetables, wholegrain bread with egg, oatmeal and wholegrain pizza. They also considered sources of protein, such as fish, chicken, meat and beans, as important components of a healthy diet. Participants considered adolescents to be more concerned about healthy eating than younger children, as illustrated by an 11-year-old girl:

*(…) it could be that they (the older children) want to have healthier food, than younger children, (…). I think that the small children only want to have sweets, because there is a long line at the kiosk after their training. I think most of them buy chocolate*. (Participant 2, focus group 3)

Participants’ motivation for healthy eating was often linked to their physical performance at trainings or competitions, as exemplified by comments from 13- and 14-year-old boys:

*Participant 4: (…) I sometimes bring sandwiches from home, because they [kiosk] don’t have that much food that is good for you prior to a match.*

*Participant 5: They offer a lot of cakes.*

*Participant 4: And sausages.*

*Participant 5: And Coke and that stuff, that you shouldn’t have prior to a match.* (Focus group 2)

### Experiences with the food selection at the sports arena

Participants in each focus group had different experiences with the selection of food available at their respective sports arenas. Overall, participants experienced limited availability of healthy food at the sports arena and asked for a healthier food selection. Most participants said that the food selection at their sports arena had food that was heavy to digest, such as sausages, waffles and burgers. Participants often mentioned that they bought their own healthy food from outside the sports arena, as explained by a 14-year-old boy: ‘*I feel that there is not much healthy food that provides you energy. (…) If you want to have food that satisfies so that you can play a good match, you should go the food store’* (Participant 5, focus group 2). When asked about what kind of healthier food they believed was missing from the selections, participants across all focus groups mentioned wraps, smoothies, fruits, juices, protein bars, warm food and a broader selection of sandwiches. However, one participant outlined that they should be satisfied with the food selection at the kiosk given its basic kitchen facilities.

The food selection at tournaments was perceived as being even more unhealthy than what was provided when they were training, as indicated in a discussion between two boys: ‘*D5: There are a lot of cakes and such stuff. D4: Sausages also. D5: Yes, and it might not be optimal before a match… maybe you should bring good food from home then’* (Participant 4, 5, focus group 2). A 12-year-old girl (focus group 3) explained that ‘*they sell a lot of sausages, waffles, muffins, slush, and candies*’ during the tournaments. Even participants who were normally pleased with the food selection would have appreciated healthier alternatives during tournaments. A 14-year-old boy explained: *‘To get food that makes you play a good match, you have to go to the food store’* (Participant 5, focus group 2). The boys from focus group 2 also told that the kiosk was not always opened during training and that there was more unhealthy food during tournaments.

Some participants from focus group 2 perceived that they were not the target audience of the sellers in the kiosks. Especially at competitions, participants felt that the food selection available at the sports arenas was targeted towards the spectators rather than the players, as illustrated by the following statement by a 14-year-old boy: *‘They should also think about the sport players’ food, not only what the spectators want’* (Participant 1, focus group 2).

### Factors influencing participants’ food choices at the sports arena

As described above, participants often did not find healthier food options at their kiosk. The limited availability of healthy options was an important reason why participants avoided buying food at the kiosk. In addition, the high price of the food, especially healthier alternatives, from the kiosk was an important factor that influenced participants food choices. The 14-year-old boys in focus group 2 explained that *‘we don’t use to buy something from the kiosk after regular trainings. We eat at home. The kiosk is too expensive’.* Participants in all focus groups mentioned high food and beverage prices as reason for reduced purchases from the kiosks. Many preferred to buy unhealthier but cheaper food at the kiosk. Others avoided to buy food at the kiosk and bought cheaper food at a nearby food store.

Parents and trainers often influenced what the participants bought at the kiosks. The following quote from a 13-year-old girl shows that participants’ parents often provided them with food before they arrived at the sports arena: *‘But parents often provide us with food from home so that we don’t have to buy anything at the tournaments. They buy food for us so that we don’t need to spend time to shop and to prepare food’* (Participant 5, focus group 2). On the other hand, participants commonly received unhealthy food as a reward from their parents. This was illustrated by a comment from a 12-year-old girl: *‘My dad sometimes says, ‘when you score three goals, you will get a chocolate’* (Participant 2, focus group 2). Two participants (focus group 4) told that their trainers motivated them to eat healthy and that the trainers often asked if they eat healthy and regularly. The trainers influence on participants’ food choices was also found in other interviews. The boys in focus group 2 explained: *‘When there is a tournament, our trainer sometimes drives to a nearby grocery store to buy some healthy food for us. (He/she) does not allow us to eat unhealthily’.*

Some participants said that their food choices at the kiosks were influenced by appearance of the food and the marketing surrounding it. A 13-year-old girl explained it as follows: *‘Mh, yes, there are some posters, and flags with marketing for Slush at the kiosk. And when you see them, … It looks better than sausages’* (Participant 2, focus group 3).

A participant from the same focus group added that she often got tempted by the unhealthy food that her teammates ate. Participants in other focus groups also admitted to being influenced by their peers’ food choices and habits. Some did not believe that anything besides appearance and taste influenced their food choices: *‘If it is good food, and it looks appealing (…) It should not look disgusting. Then you don’t want to eat it’* (Participant 4, focus group 1).

### Expectations related to a healthy food selection at the sports arena

Throughout the interviews, participants stressed the importance of a healthy food selection and their expectations for the same at the sports arena, as illustrated by two 13–14 years old boys: D4: ‘*It would be important to have nutritious food, maybe with carbohydrates prior to a competition*’. D5: ‘*food that gives you energy for a long time*’ (Participant 4 and 5, focus group 2). Interestingly, two girls in focus group 4 (13–14 years old) believed that the kiosk also should offer unhealthy food so that *‘we do not feel the forced to buy only healthy food*’.

Participants discussed that the club managers and competition organisers were responsible for providing healthy food selections at the sports arenas. However, participants thought that the managers needed to provide unhealthy foods to turn a profit, as illustrated by a 14-year-old boy: *‘Yes, most of the kiosks think about their income (…) This is why they sell mostly unhealthy food. It’s understandable’* (Participant 1, focus group 2).

## Discussion

Adolescents across the focus groups perceived the food selection available at the sports arenas as unhealthy. Price, marketing and availability of unhealthy food were important factors that influenced their food choices at the sports arena. Several studies have investigated the factors influencing adolescents’ food choices^(13,24,25)^.

Cost appeared to be an important factor driving adolescents’ food selections at the sports arena. Several participants admitted to buying less or no food at the sports arena due to the high prices. They also experienced that unhealthy food often was cheaper than healthier alternatives at the kiosk. Cost was identified as an important factor in adolescent food choices outside the home in other studies as well^(24,26)^. However, food and beverage sales are an important source of income for the sport clubs^(16,18)^.

Participants in our study were often influenced by the marketing of unhealthy food. Sport is a very powerful avenue that creates identity, unity and community, and it has a wide impact through the values and messages it promotes. Sponsorships by brands selling unhealthy foods are problematic in sports arenas^(16,27–30)^. Studies in Australia, the USA and New Zealand revealed that sponsorship from unhealthy food producers was a barrier for healthy food provision^(31–33)^. Thus, sponsorship of healthy food might be an important facilitator for a healthier food selection at the sports arena. Cultural changes, such as widespread professional guidelines in clubs, preferably by the national federation with support from elite athletes who promote healthy food ideals, could enforce a healthy food identity in the sports context^(16,28,30)^. As such, the steps taken by the Norwegian parliament to forbid the marketing of unhealthy foods towards children, and to put an age restriction for the sale of energy drinks, are important political measures that can pave the way for similar initiatives by other nations. Taste or physical properties of food may be important factors affecting food choices among adolescents^(25,34)^. Participants in our study reported to be tempted by the unhealthy food that their teammates ate. However, as this study was conducted among active adolescents who frequently played sport, their food choices was mainly motivated by health and performance factors.

Adolescents in this study were interested in healthy eating and asked for a healthier selection of food at the sports arena. Even though participants in this study would prefer healthy food selections to be available at sports arenas, our findings are in line with other national and international studies with parents and club managers that characterise the food selection at sports arenas as being unhealthy^(19)^. For instance, club managers and parents at Norwegian handball and football clubs described kiosks selling a predominantly unhealthy selection of food. However, interviews among parents revealed an ambivalence as some also appreciated the availability of unhealthy food at the sports arena^(19)^. Parents play an important role in creating and supporting the healthy dietary habits of adolescents^(35,36)^. Adolescents in the focus groups mentioned that their parents often gave them unhealthy food at the sports arena as a reward for playing well. Using food as a reward has been associated with emotional overeating in a population-based cohort in the Netherlands^(37)^. Hence, providing unhealthy food as a reward in the sports context may negatively influence adolescents’ future dietary behaviour. Parents in a focus group study in Minnesota were also less concerned about the importance of healthy food than their children were^(14)^.

Several adolescents were motivated by their trainers to eat healthy. We have not found any studies that specifically investigate the trainers’ roles in adolescent food consumption in sports settings. However, a review of the effectiveness of family-based and institutional interventions in improving children’s diets determined that school-based programmes should incorporate role models, such as peers, teachers and heroic figures to improve effectiveness, in addition to rewards and increased access to healthy foods^(38)^. Thus, trainers may have an important role in promoting healthy food choices at the sports arena.

## Study limitations

This study was conducted with a small sample size, which is typical of qualitative studies^(23)^. Nonetheless, we believe that our findings may be transferable to any sport arena targeting adolescents of similar sizes with comparable user groups, economies and facilities. All of the authors were female and had personal experiences with either being active, or having active children, at Norwegian sport arenas. Thus, the researchers’ personal experiences and professional backgrounds might have shaped the gathering and interpretation of the data. Interestingly, even though the participants in each focus group varied in terms of gender and the size of the sports club they represented, we did observe informational power related to the aim of this study^(21)^. However, interviewing a more diverse range of adolescents, in terms of sports played, their locations or their demographic backgrounds, might have revealed additional themes.

### Conclusions

Adolescents wished for a healthier selection of food at sports arenas. Cost of food emerged as a factor that influenced their food choices. In addition, introducing substitutions of healthier alternatives may influence the attempt to make a healthier food choice. Our study also indicates that marketing of unhealthy food and beverages should be restricted, to influence adolescence food choice towards healthier alternatives. More insights into the food choices in other parts of Norway and in other types of sports are highly warranted to promote and stimulate positive healthy behaviours among adolescents at the sports arena.
